# Cognitive flexibility in younger and older children who stutter

**DOI:** 10.3389/fpsyg.2022.1017319

**Published:** 2022-11-18

**Authors:** Maria Paphiti, Kurt Eggers

**Affiliations:** ^1^Department of Psychology and Speech-Language Pathology, University of Turku, Turku, Finland; ^2^Department of Rehabilitation Sciences, Speech-language Pathology/Audiology Research Group, Ghent University, Ghent, Belgium; ^3^Department of Speech-Language Therapy and Audiology, Thomas More University College, Antwerp, Belgium

**Keywords:** stuttering, set-shifting, cognitive flexibility, mixing-cost, set-shifting-cost, executive function

## Abstract

**Purpose:**

Recent research findings suggest possible weaknesses in cognitive flexibility (*CF*) in children who stutter (CWS) when compared to children who do not stutter (CWNS). Studies so far, have been conducted with either younger (3–6 years old) or older children (6–12 years old) with a variety of measures. The purpose of the present study was to investigate *CF* with the use of a single behavioral measure across a broader age range (4–10 years old).

**Methods:**

Participants were 37 CWS (mean age = 6.90 years) and 37 age-and gender-matched CWNS (mean age = 6.88 years), divided in a younger (below 7 years) and older (above 7 years) age group. All participants undertook a computerized visual set-shifting task consisting of three blocks. *CF* was evaluated through across-and within-block comparisons of the actual response speed and accuracy values. In addition, mixing-and set-shifting-costs were evaluated based on the mean response speed and accuracy.

**Results:**

All participants showed expected mixing-and set-shifting-costs. Only the within-block analyses yielded significant between (sub)group differences. Investigation of the block × classification group × age group interactions showed that older CWS had larger set-shifting-costs (slowed down more and made more errors) compared to older CWNS.

**Conclusion:**

While all participants required more time during set-shifting trials, only the older CWS (7–10 years old), and not younger CWS, were slower and made more errors. This finding corroborates previous findings in CWS of a similar age and could possibly point to a role of *CF* in stuttering persistence.

## Introduction

Developmental stuttering is a complex neurodevelopmental and multifactorial disorder which usually first appears between the ages of 2.5 and 4 years and is characterized by sound-, syllable-, monosyllabic word-repetitions, blocks, broken words and sound prolongations (Ambrose and Yairi, 1999; Yaruss and Quesal, 2004). Beyond the core behaviors, stuttering may be accompanied by secondary physical behaviors such as facial grimaces or movements of the extremities as well as cognitive and emotional reactions such as fear/avoidance of speaking in certain communicative situations (Guitar, 2014, p. 24). Most children outgrow developmental stuttering with or without therapeutic intervention. The lifetime incidence in the general population is about 8% and lifespan prevalence is around 0.7% (Yairi and Ambrose, 2013). Due to the cognitive and emotional reactions to disfluencies, stuttering may negatively affect the quality of life of people who stutter (Hayhow et al., 2002; Klein and Hood, 2004; Craig et al., 2009).

Over the years, several theoretical models have been developed in an attempt to explain the appearance and/or persistence of stuttering. Some of these models specifically refer to possible weaknesses in cognitive flexibility. The Vicious Circle Hypothesis (Vasić and Wijnen, 2005) for example, suggests that the attention parameters (i.e., effort, focus, and threshold) of the internal monitoring system for detecting and correcting errors prior to articulation are inappropriately set in people who stutter (Vasić and Wijnen, 2005, p. 233), making it possible that weaknesses in attentional/cognitive flexibility exacerbate the production of disfluencies (Oomen and Postma, 2001). A more recent model, the Executive Function Model of Developmental Stuttering (Anderson and Ofoe, 2019), suggests that weaknesses in the EF development (i.e., working memory, inhibitory control and cognitive flexibility (*CF*; Pennington and Ozonoff, 1996; Miyake et al., 2000; Zelazo and Müller, 2011; Cragg and Chevalier, 2012; Diamond, 2013) may be associated with weaknesses in domain-specific processes (i.e., linguistic, motor, sensory and emotional) that have been found to be associated with the appearance and persistence of developmental stuttering.

In the literature, *CF* has been used interchangeably with the terms set-shifting, attention shifting, switching, and attentional flexibility (Ionescu, 2012). Despite the different terms used, researchers agree that *CF* skills allow us to adapt easily to changes in the environment (Moriguchi and Hiraki, 2009). *CF* refers to: (a) being able to stabilize attention to what is relevant and ignore what is not relevant (Benitez et al., 2017), that is, to view things from a different perspective, to complete tasks that involve sorting things based on two or more different dimensions (e.g., color and shape), also known as *switching*, as well as (b) being able to shift attention accordingly to the changing demands, that is, to learn a rule and subsequently shift to the newly introduced one, to make transitions, to adapt to changes in the environment (Moriguchi and Hiraki, 2009), also known as *set-shifting* (Garon et al., 2008; Nigg, 2017).

The development of *CF* starts in early preschool years (Luciana and Nelson, 1998), with significant changes appearing as early as late preschool years (5–6 years; Moriguchi and Hiraki, 2009), and continues until early adolescence (Cepeda et al., 2001; Anderson, 2002; Huizinga et al., 2006). Switching develops between the ages of 3–5 years (Doebel and Zelazo, 2015), while set-shifting is reported to develop somewhat later on and to mature rapidly between the ages of 8–10 years (Klimkeit et al., 2004).The two domains of *CF*, switching and set-shifting, build upon the other two EFs and that is why *CF* tasks in general, often tap onto working memory and inhibitory control. Due to the increase in complexity, *CF* “can be seen as an EF process operating on another EF process” (Garon et al., 2008, p. 50).

There are different ways to evaluate *CF*, ranging from parental questionnaires to performance tests such as (non-)computerized behavioral tasks. In the first type, parents are asked to rate their child’s behavior on different EF scales, while in the second, children are asked to complete different tasks and their performance is measured usually based on response time, number of errors, number of failures and number of corrections given by the examiner.

There are two types of computer paradigms that are frequently used to measure *CF*: (a) Alternation-design paradigms for measuring the domain of switching and (b) Mixed-block-design paradigms for measuring the domain of set-shifting (Gopher et al., 2000). Alternation-design paradigms consist of two phases: a pre-switch and a post-switch phase. In these paradigms, participants are usually required to sort test cards based on one dimension during the pre-switch stage (e.g., color), and based on another dimension (e.g., shape), during the post-switch stage. The dimension change during a switch results in slower and/or less accurate responses, i.e., performance-cost (Eichorn and Pirutinsky, 2021).

Mixed-block-design paradigms include repeated presentations of the same task (i.e., initially following one rule and then following another one), as well as shifts between tasks within the same block, (i.e., set-shifting between the two rules). Some of these paradigms include three blocks, as in the present study. The first block is compatible (responding to the side of stimulus presentation), the second is incompatible, while the third is mixed (either compatible or incompatible stimulus is presented). Mixed-block-design paradigms allow researchers to evaluate performance-cost in two ways: (a) by measuring the difference in speed and/or accuracy between the compatible block and the compatible trials of the mixed block, known as mixing-cost (Reimers and Maylor, 2005; Cragg and Chevalier, 2012) and, (b) by measuring the difference in speed and/or accuracy between the no set-shifting and the set-shifting trials within the mixed block, known as set-shifting-cost (Schmitter-Edgecombe and Langill, 2006). When measuring mixing-cost, an across-block comparison is conducted, whereas when measuring set-shifting-cost, a within-block comparison is conducted.

Only eight studies investigated *CF* in CWS and CWNS, with all of them suggesting possible associations between *CF* weaknesses and developmental stuttering (Eggers et al., 2010; Eggers and Jansson-Verkasalo, 2017; Eichorn et al., 2018; Ntourou et al., 2018; Rocha et al., 2019; Anderson et al., 2020; Eichorn and Pirutinsky, 2021; Paphiti et al., 2022). Two used parental questionnaires (Eggers et al., 2010; Ntourou et al., 2018), two used non-computerized behavioral tasks (Ntourou et al., 2018; Rocha et al., 2019), and five used behavioral computer tasks (Eggers and Jansson-Verkasalo, 2017; Eichorn et al., 2018; Anderson et al., 2020; Eichorn and Pirutinsky, 2021; Paphiti et al., 2022). Both Ntourou et al. (2018) and Eggers et al. (2010), the first using the Behavioral Rating Inventory of Executive Function-Preschool (BRIEF-P; Gioia et al., 2003) and the latter the Children’s Behavior Questionnaire-Dutch (Van den Bergh and Ackx, 2003), reported weaknesses in shifting. Rocha et al. (2019) was the only cross-sectional study conducted. The Children’s Color Trail Test (CCTT; Pinto, 2008) was used which is a paper-and-pencil test. The researchers reported that the younger subgroup of CWS required longer response times and made more errors than their age-and gender-matched CWNS. These *CF* weaknesses for the 7–9-years-old CWS subgroup but not for the 10–12-years-old seems to suggest that *CF* develops slower in CWS but reaches similar performance around the age of 10. In the five behavioral studies that used only computer tasks, *CF* was evaluated by measuring the speed and accuracy of the participants’ responses. In three of the studies, alternation-design tasks were used, assessing the *CF* domain of switching (from one dimension to another; Eichorn et al., 2018; Anderson et al., 2020; Eichorn and Pirutinsky, 2021), and in two, mixed-block-design tasks were used, assessing the *CF* domain of set-shifting (between two already introduced rules; Eggers and Jansson-Verkasalo, 2017; Paphiti et al., 2022). All reported weaknesses in *CF* for CWS.

Taken all together, the current findings do not provide us with sufficient information as to how *CF* develops in the two groups, since most of these studies included either younger (3–6 years old) or older (6–12 years old) children. So far, there are few indications that the development of *CF* may be different between CWS and CWNS. There is no information though, about the development of *CF* in CWS from preschool to early school-age years.

Therefore, the aim of the present study was to assess *CF*, more specifically set-shifting, within and across two classification groups (CWS and CWNS) and two age groups (younger and older). By having a wider age range (4–10 years) compared to that of previous studies, we were able to divide both classification groups into younger (CWS-Y and CWNS-Y: below 7 years; preschool-age children) and older subgroups (CWS-O and CWNS-O: above 7 years; school-age children) and conduct a cross-sectional study. This was to provide us with information as to how *CF* develops across this wider age-range. To measure *CF*, we did not only compare the compatible trials but, we also compared the incompatible trials. Even though, the standard measure of *CF* suggests comparing the compatible trials, we proceeded with also comparing the incompatible trials as an attempt to control for the effects of inhibitory control.

The main research questions were:

1. Are CWS, as a group, slower and/or less accurate than CWNS in a *CF* task?
2. Are CWS-Y and CWS-O, slower and/or less accurate than, respectively, CWNS-Y and CWNS-O in a *CF* task?

In all the analyses, we hypothesized that there would be significant differences expressed in longer response times but not in more errors for the subgroups of CWS-Y, CWS-O and for the group of CWS. This finding is hypothesized to be more evident in the analyses of incompatible trials as it was earlier reported (Paphiti et al., 2022).

## Materials and methods

### Participants

Participants were 74 Dutch-speaking children (56 boys and 18 girls: 37 CWS and 37 CWNS). The mean age for the CWS was 6.90 years (SD = 1.37 years; age range: 4.08–9.50), and for the CWNS was 6.88 years (SD = 1.40 years; age range: 4.00–9.33), *t*(72) = −0.06, *p* = 0.95. CWS and CWNS were age-(±3 months) and gender-matched. Participants were divided based on their age into CWS-Y and CWNS-Y (below the age of 7 years old) and CWS-O and CWNS-O (above the age of 7 years old). Table 1 shows the demographic characteristics of these four subgroups.

**Table 1:** Demographic characteristics of the participants in the 4 subgroups (children who stutter-younger and older = 37; children who do not stutter-younger and older = 37).

Group	CWS-Y	CWS-O	CWNS-Y	CWNS-O
*n*	19	18	18	19
Age range	4.08–6.92	7.08–9.50	4.00–6.75	7.08–9.33
Age mean (SD)	5.82 (0.82)	8.04 (0.75)	5.69 (0.81)	8.00 (0.74)
Sex (M/F)	14/5	14/4	13/5	15/4

All participants attended mainstream pre-school or elementary school. Data concerning the clinical profile of the participants and the educational level of the parents were collected using parental questionnaires. The questionnaires revealed that there was no parental concern regarding stuttering (except for the CWS), none of the participants had any known psychological, neurological, visual, or developmental problems and no history of previous speech and/or language therapy (except for stuttering therapy for CWS). Two CWS and 3 CWNS were reported as left-handed, while all other participants as right-handed. The parental educational level was based on the combined scores of the highest level completed of each family (primary education = 1, high school = 2, Bachelor’s degree = 3, Master’s degree = 4), as done in previous publications (Eggers et al., 2013, 2018). No significant group differences were found (mean = 5.65, SD = 1.11 for the CWS group; mean = 6.11, SD = 1.35 for the CWNS, *U* = 543.50, *p* = 0.12).

In order to exclude any possible cognitive group differences, two subtests of the Wechsler Intelligence Scale for Children-Third Edition (WISC-3; Wechsler, 2005)—Vocabulary and Block Design Subtests—were administered. The WISC-3 consists of seven performance and six verbal subtests. The two abovementioned subtests were chosen because they correlate well with the overall score of the test (Groth-Marnat, 1997). In the Vocabulary subtest, participants were asked to explain the meaning of single words, while in the Block Design subtest, they were asked to rebuild a geometrical pattern with the use of 4 to 9 two-colored cubes (as quickly and as correctly as possible). Mean Vocabulary score was 20.22 (SD = 7.71) for the CWS and 20.54 (SD = 9.12) for the CWNS. Mean Block Design score was 25.70 (SD = 13.70) for the CWS and 28.97 (SD = 14.79) for the CWNS. No significant group differences were identified either in the Vocabulary subtest, *t*(72) = 0.17, *p* = 0.87, nor in the Block Design subtest, *U* = 579.50, *p* = 0.26.

In order to assess the language skills of the participants, two subtests (Vocabulary Production and Sentence Production) of the Language Test for Children (Van Bon and Hoekstra, 1982) were administered. In the Vocabulary Production subtest, participants were presented with a picture, and they were asked to complete a sentence with the target-word; in the Sentence Production subtest, participants were expected to correct the syntactical errors in the phrases presented to them. Normal language function is considered a score above percentile pc16 in both subtests. The mean percentiles for the CWS on the Vocabulary Production subtest were 63 (range 27–98), while for the CWNS they were 72 (range 27–97). The mean percentiles for the CWS on the Sentence Production subtest were 55 (range 35–99), while for the CWNS they were 63 (range 27–99). In order to assess children’s articulation, the Antwerp Screening Instrument for Articulation (ASIA-5; Stes and Elen, 1992) was administered. This screening test requires from children to produce age-appropriate phonemes in various word positions. All children included in the study, produced age-appropriate phonemes as required by the test.

Participants’ hearing was screened at 500, 1,000, 2,000, and 4,000 Hz with the use of the Accuscreen (Wood, 2003), a handheld hearing-screening device with signals presented at 20 db. All participants passed the hearing screening.

Two spontaneous speech samples were collected from all participants on 2 different days. Scores on the Stuttering Severity Instrument-3 (SSI-3; Riley, 1994) were based on a minimum of 300 words. CWS produced at least three monosyllabic word repetitions and/or within-word disfluencies (sound/syllable repetition, prolongation, broken words or blocks) in 100 words of spontaneous speech (Conture, 2001). Five CWS were classified as very mild, 17 as mild, 12 as moderate, two as severe and one as very severe.

### Materials

#### Baseline speed task

The baseline speed task (De Sonneville, 2009) is a simple response time computer paradigm. Participants undertook this task prior to undertaking the computer paradigm used for this study. It was administered to get participants acquainted with computerized testing and to minimize the possibility of any response time differences that might confound the results of the experimental task. Participants had to fixate on a white cross in the center of a black laptop screen. They were instructed to press the response key as soon as the signal changed into a white centralized square. The task consists of two blocks of trials. In one of the blocks, participants pressed the response key only with their left index finger, while in the other, they pressed it with only their right index finger. Right-handed participants began the task by pressing the response key with the left index finger, while left-handed participants began the task with the right index finger. Prior to these two experimental blocks of 32 trials each, participants watched an instruct session of two trials and completed a practice session of ten trials. Signal duration was variable until a response was given. For a response to be considered valid, it had to fall between 150 and 4,000 ms after stimulus onset. Post response intervals varied randomly from 500 to 2,500 ms.

#### Experimental computer paradigm: Set-shifting visual task (SSV)

**Figure 1:**
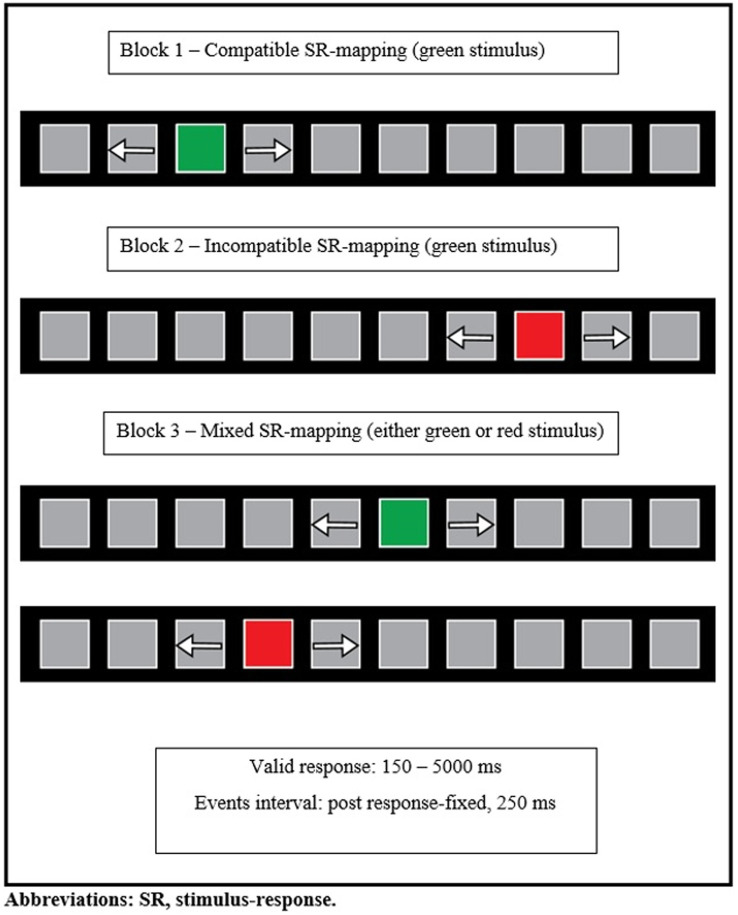
Schematic overview of the shifting attentional set-visual task.

The Set-Shifting Visual task (SSV) is part of the Amsterdam Neuropsychological Tasks (ANT; De Sonneville, 2009), and evaluates *CF* by measuring the speed and accuracy of manual responses of the participants. It consists of three blocks. The stimulus is a green-or red-colored square that changes position on a permanently present horizontal bar of 10 squares on a black computer screen. The colored square jumps randomly either to the left or to the right of the horizontal bar (Figure 1). The green square indicates a compatible trial (Block 1-compatible block), while the red indicates an incompatible trial (Block 2-incompatible block). In the compatible block, participants were instructed to respond in a “spatially compatible way” (Serlier-van den Bergh, 2002, p. 156), i.e., they were instructed to press the right key with the right index finger if the green-colored square appeared on the right side of the horizontal bar; to press the left key with the left index finger if the green-colored square appeared on the left side of the horizontal bar. In the incompatible block, participants were instructed to press the opposite key as soon as the red-colored square moved. In the mixed block, the stimulus–response mapping is mixed. Both compatible and incompatible trials appear in random order. Participants were instructed to press the corresponding response key if a green-colored square appeared, or the opposite response key if a red-colored square appeared. Valid responses (correct or error) had to fall between 150 and 5,000 ms after stimulus onset. Any premature responses or omissions were removed from the data. Post response intervals were fixed at 250 ms. No feedback was provided. The compatible and incompatible blocks consist of 40 trials while the mixed block consists of 80 trials. Prior to each experimental block (for the compatible and incompatible blocks), an instruction session (6 trials) followed by a practice session (10 trials) were conducted. In the mixed block, the instruction session had five, while the practice session had 15 trials. In the mixed block, there was “random alteration of stimulus response mapping conditions” (Serlier-van den Bergh, 2002, p. 157), asking participants to set-shift their attention between the two previously applied rules (compatible and incompatible stimulus–response mapping conditions). In this block, there were 40 set-shifting trials (20 set-shifts from compatible to incompatible and 20 set-shifts from incompatible to compatible). The task data were recorded and stored automatically by the task software for each participant, as mean response times and total number of errors per block. Additionally, we manually extracted the response time and error value for each individual trial of each participant in each block and used these values for our analyses.

As per the task manual (De Sonneville, 2009), *CF* was determined by comparing the measurements of speed and accuracy in the compatible block with those of the compatible trials of the mixed block. To control for the effect of inhibitory control, we also compared the speed and accuracy of the trials from the incompatible block to the incompatible ones of the mixed block. In case of an interruption between trials, trials after the interruption were excluded from these analyses.

### Procedure

All participants were paid volunteers. The CWS were recruited through their speech-language pathologist, while the CWNS were recruited through their school. All data were collected in a quiet setting at the home of the participant by the second author. The baseline speed and the SSV tasks were presented on a laptop with a 15–in. screen. Participants were sat facing the laptop that was placed on a table in front of a plain wall (approximately 18 inches from them). To avoid distracting visual stimuli, a large black pliable cardboard was positioned around the laptop, and participants wore noise-reducing headphones to minimize possible distracting environmental sounds. Data collection required two 45-min sessions (sessions A and B) and tests within each session were always administered in the same order. In session A, the first speech sample was collected during a play activity; the speech-language testing and the hearing screening were also performed. In session B, both the baseline speed and the SSV task were administered followed by the two subtests of the WISC-3 and the second speech sample. Data collection for half of the participants began with session A, while for the rest it began with session B, to minimize the possibility of test-order-confound. None of the procedures used in this study were invasive and the study was approved by the Research Ethics Committee of Leuven University Hospitals. All parents agreed for their child’s participation by signing an informed consent form.

### Data analyses

A Mann–Whitney test was used to determine whether there were any response time differences between the two classification groups in the baseline speed task to avoid any interference with the results of the SSV task. This test was selected because the scores of the two groups were not normally distributed.

Cognitive flexibility was investigated by comparing speed and accuracy of (a) the trials of the compatible block with the compatible trials of the mixed block (Block 1 vs. Block 3 compatible trials) and (b) the trials of the incompatible block with the incompatible trials of the mixed block (Block 2 vs. Block 3 incompatible trials). For investigating speed, a gamma generalized linear mixed model was conducted with response times as the dependent variable. This model allows the investigation of the multilevel experimental design, and is particularly suited for positively-skewed non-negative data (Dean and Nielsen, 2007). The model included: (a) fixed effects for (i) block with three levels (for the compatible analysis: compatible block, compatible trials of the mixed block with set-shifting, compatible trials of the mixed block with no set-shifting; for the incompatible analysis: incompatible block, incompatible trials of the mixed block with set-shifting, incompatible trials of the mixed block with no set-shifting), (ii) classification group (CWS vs. CWNS), and (iii) age group (younger vs. older); (b) all two-and three-way interactions between these variables; and (c) random intercepts for each subject.

For investigating accuracy, a binomial generalized linear mixed model was performed in both analyses, with error (correct vs. incorrect response per trial) being the dependent dichotomous variable. The model included the same fixed effects, interactions, and random intercepts as the speed analyses. Based on our hypotheses, the main effects and interactions in the multilevel regression analyses were planned. The significance level for all analyses was *α* = 0.05. Data analysis was conducted using SPSS (Statistical Package for the Social Sciences—Version 25 for Windows, IBM, Corp., Armonk, NY, United States).

## Results

No significant differences in response times between the two classification groups were found in the baseline speed task, *U* = 623.50, *p* = 0.51. The mean response times for the CWS were 455 ms (SD *=* 136) and 476 ms (SD *=* 153) for the CWNS.

### Cognitive flexibility in terms of speed

#### Compatible trials

The coefficients for the multilevel regression analysis are shown in Table 2. The between-subjects effect for the classification group factor was not significant, *F*(1, 5,908) = 2.37, *p* = 0.12 (for CWS: *M* = 1,150 ms, SE = 41 ms; for CWNS: *M* = 1,242 ms, SE = 44 ms). The effect of age group was significant, *F*(1, 5,908) = 13.10, *p* < 0.001 (for younger participants: *M* = 1,309 ms, SE = 45 ms; for older participants: *M* = 1,091 ms, SE = 40 ms). The only significant interaction was the one between age group and block, *F*(2, 5,908) = 34.92, *p* < 0.001. Investigation of this interaction revealed that both age groups had an increase in response times (i.e., mixing-cost) moving from the compatible block to the mixed block, both for the set-shifting and no set-shifting trials (*p* < 0.001 in all cases); in addition, both age groups had significantly longer response times for set-shifting trials compared to no set-shifting trials (for younger participants: *t*(5908) = 10.08, *p* < 0.001; for older participants: *t*(5908) = 9.29, *p* < 0.001). Moreover, the two age groups differed in the compatible block, *t*(5908) = −6.45, *p* < 0.001, but not in the mixed block (for the set-shifting trials: *t*(5908) = −1.85, *p* = 0.06); for the no set-shifting trials: *t*(5908) = −1.70, *p* = 0.09. No other interaction was significant.

**Table 2:** Fixed effects of classification group, age group and block on response times (compatible trials analysis).

Model term	*B*	SE	*t*	*p*	95% CI for Exp(*B*)	
					LL	UL
Intercept	6.93	0.05	131.76	< 0.001	6.83	7.03
classification group (CWS)	−0.06	0.07	−0.77	0.44	−0.20	0.09
Block (mixed-set-shifting)	0.54	0.04	15.29	< 0.001	0.47	0.61
Block (mixed-no set-shifting)	0.25	0.04	7.00	< 0.001	0.18	0.31
Age group (older)	−0.29	0.08	−3.89	< 0.001	−0.44	−0.15
Classification group (CWS) × Block (mixed-set-shifting)	0.03	0.05	0.63	0.53	−0.07	0.13
Classification group (CWS) × Block (mixed-no set-shifting)	0.05	0.05	1.04	0.30	−0.05	0.15
Classification group (CWS) × age group (older)	−0.11	0.11	−1.06	0.29	−0.32	0.10
Mixed block (set-shifting) × age group (older)	0.21	0.05	4.14	< 0.001	0.11	0.31
Mixed block (no set-shifting) × age group (older)	0.27	0.05	5.36	< 0.001	0.17	0.37
Classification group (CWS) × Block (mixed-set-shifting) × age group (older)	0.08	0.07	1.09	0.28	−0.06	0.22
Classification group (CWS) × Block (mixed-no set-shifting) × age group (older)	−0.03	0.07	−0.35	0.73	−0.16	0.11

The model included the random effect of an individual-level intercept. CI = Confidence Interval; LL = lower limit; UL = upper limit.

**Figure 2:**
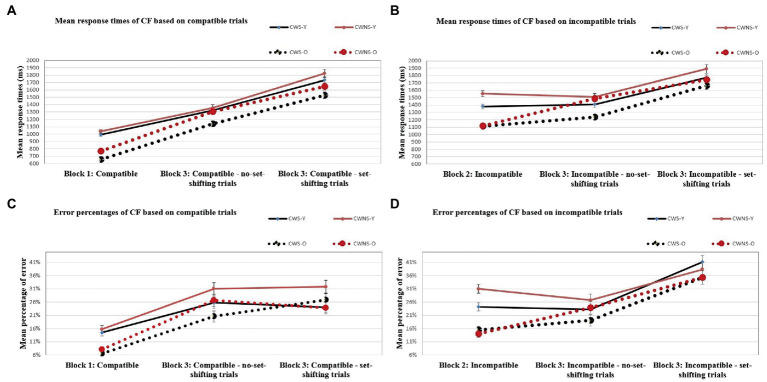
Mean response times (in ms) and error percentages of cognitive flexibility based on compatible trials (**A** and **C**), and mean response times (in ms) and error percentages of cognitive flexibility based on incompatible trials (**B** and **D**) for the CWS-Y, CWS-O and CWNS-Y and CWNS-O subgroups.

**Figure 3:**
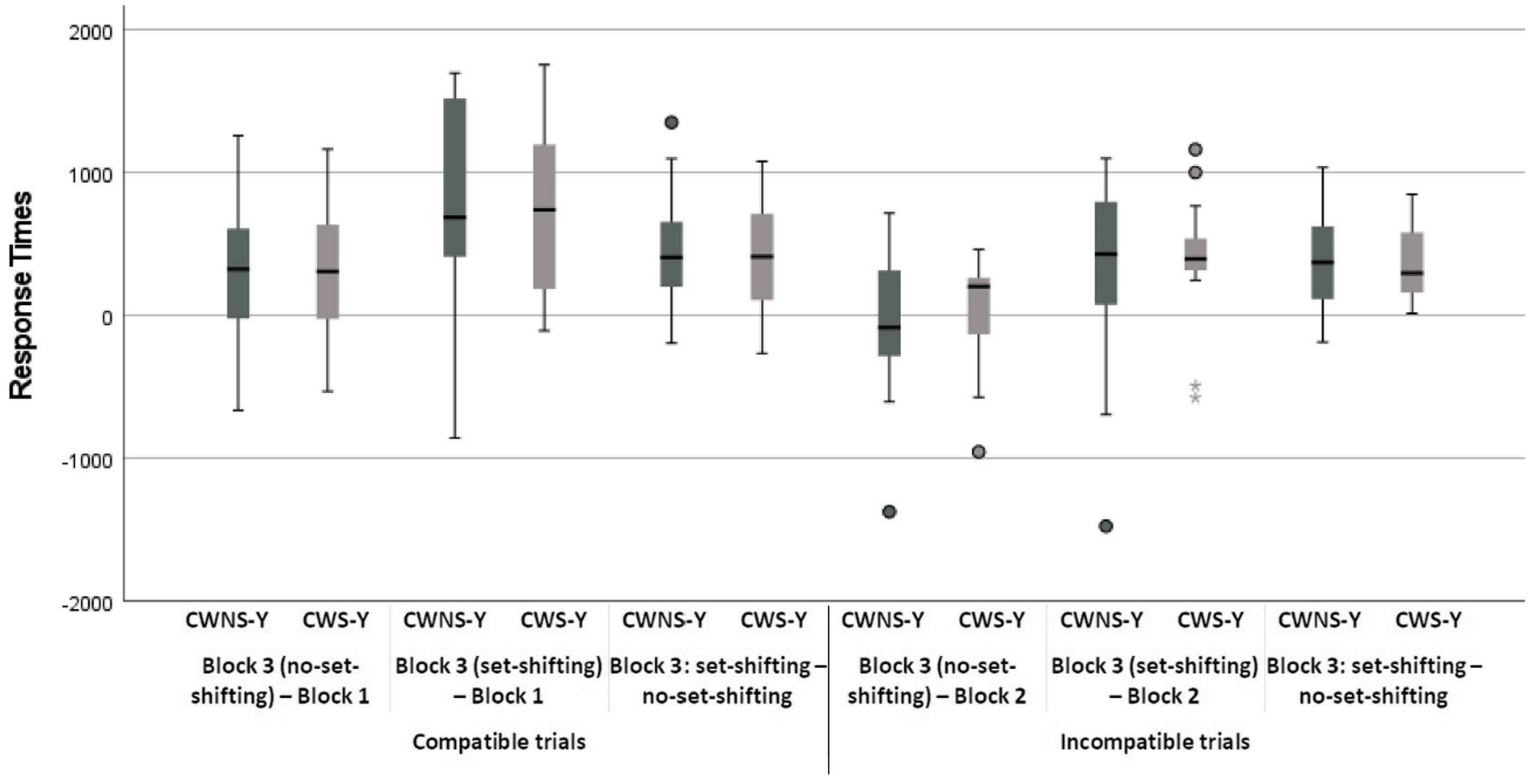
Boxplots of costs in response times for CWS-Y and CWNS-Y.

**Figure 4:**
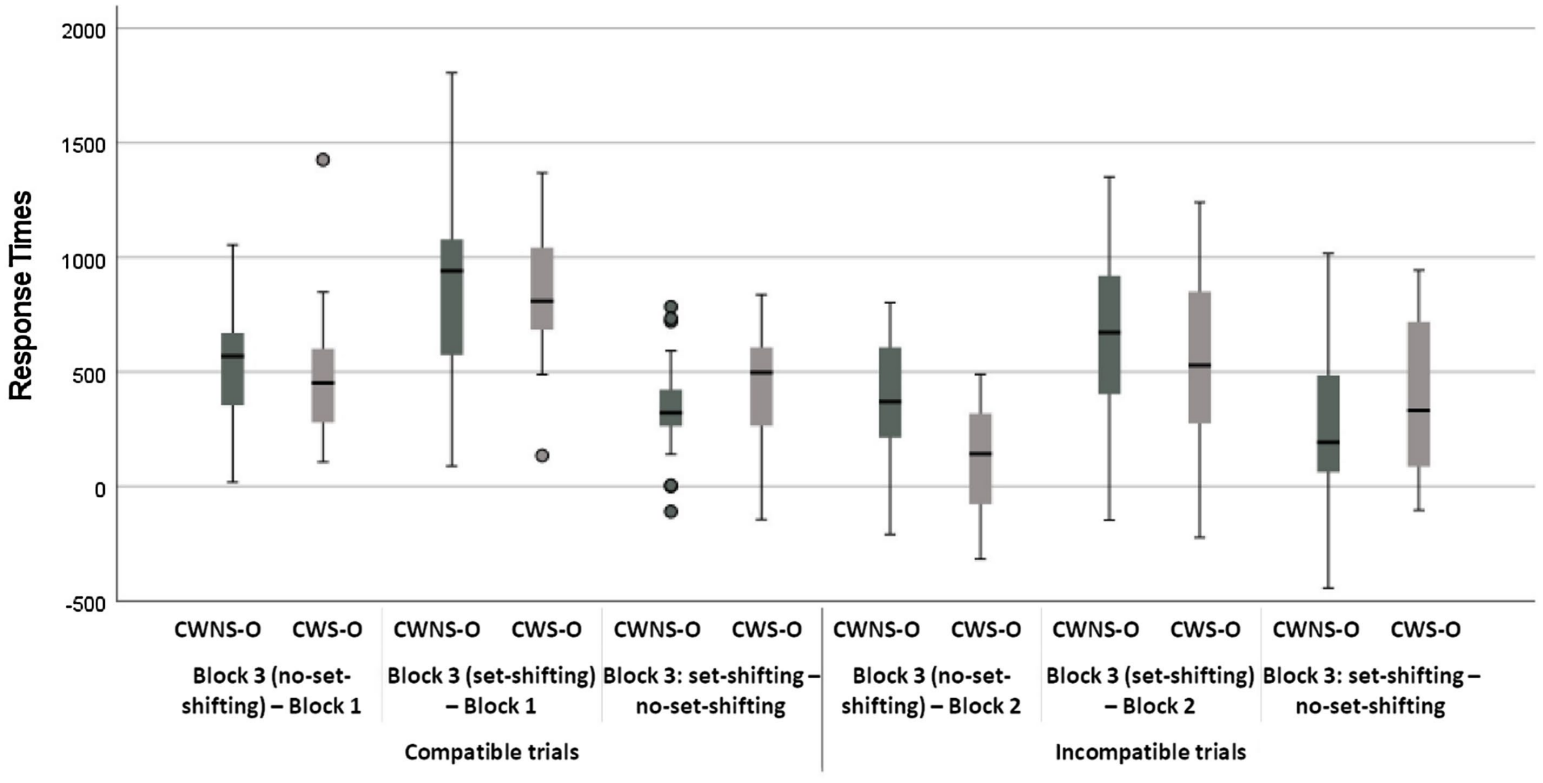
Boxplots of costs in response times for CWNS-O and CWS-O.

Nonetheless, the block × classification group × age group interaction was investigated as it was of interest. The investigation revealed that, for the set-shifting trials, CWS-O did not differ from CWS-Y, *t*(5908) = −1.54, *p* = 0.12, whereas they differed for the no set-shifting trials, *t*(5908) = −2.09, *p* = 0.04 (were faster). The CWNS-O did not differ from the CWNS-Y for either the set-shifting *t*(5908) = −1.08, *p* = 0.28, or the no set-shifting trials, *t*(5908) = −0.31, *p* = 0.76 (Figure 2, Panel A). Moreover, the investigation revealed that there were significant mixing-costs for all combinations of the age group and classification group factors (*p < 0.*001 in all cases). Two-tailed unpaired *t*-test analyses revealed that the difference in mixing-cost between any two subgroups was not significant (Figures 3, 4).

#### Incompatible trials

The coefficients for the multilevel regression analysis are shown in Table 3. The between-subjects effect for the classification group factor was not significant, *F*(1, 5,908) = 1.20, *p* = 0.27 (for CWS: *M* = 1,373 ms, SE = 62 ms; for CWNS: *M* = 1,473 ms, SE = 66 ms). The effect of age group was significant, *F*(1, 5,908) = 4.47, *p* = 0.03 (for younger participants: *M* = 1,521 ms, SE = 67 ms; for older participants: *M* = 1,329 ms, SE = 62 ms). The age group × block interaction was significant, *F*(2, 5,908) = 24.88, *p* < 0.001. Investigation of this interaction revealed that both age groups had an increase in response times (i.e., mixing-cost) moving from the incompatible block to the mixed block, both for the set-shifting and no set-shifting trials (*p* < 0.001 in all cases; the only exception was the case of the difference between the mixed block no set-shifting trials and the incompatible block for the younger participants, *p* = 0.84); in addition, both age groups had significantly longer response times for set-shifting trials compared to no set-shifting trials (for younger participants: *t*(5908) = 7.96, *p* < 0.001; for older participants: *t*(5908) = 7.75, *p* < 0.001). Moreover, the two age groups differed in the incompatible block, *t*(5908) = −4.11, *p* < 0.001, but not in the mixed block (for the set-shifting trials: *t*(5908) = −0.91, *p* = 0.36; for the no set-shifting trials: *t*(5908) = −1.04, *p* = 0.30).

**Table 3:** Fixed effects of classification group, age group and block on response times (incompatible trials analysis).

Model term	*B*	SE	*t*	*p*	95% CI for Exp(*B*)	
					LL	UL
Intercept	7.29	0.07	112.04	< 0.001	7.16	7.42
classification group (CWS)	−0.09	0.09	−0.94	0.35	−0.26	0.09
Block (mixed-set-shifting)	0.22	0.04	5.99	< 0.001	0.15	0.29
Block (mixed-no set-shifting)	−0.01	0.03	−0.40	0.69	−0.08	0.05
age group (older)	−0.30	0.09	−3.26	< 0.005	−0.49	−0.12
Classification group (CWS) × Block (mixed-set-shifting)	0.04	0.05	0.82	0.41	−0.06	0.14
Classification group (CWS) × Block (mixed-no set-shifting)	0.04	0.05	0.78	0.43	−0.06	0.13
Classification group (CWS) × age group (older)	0.06	0.13	0.46	0.65	−0.20	0.32
Block (Mimed-set-shifting) × age group (older)	0.24	0.05	4.66	< 0.001	0.14	0.34
Block (mixed-no set-shifting) × age group (older)	0.30	0.05	6.21	< 0.001	0.20	0.39
Classification group (CWS) × Block (mixed-set-shifting) × age group (older)	−0.06	0.07	−0.77	0.44	−0.20	0.09
Classification group (CWS) × Block (mixed-no set-shifting) × age group (older)	−0.19	0.07	−2.79	0.01	−0.32	−0.06

The model included the random effect of an individual-level intercept. CI = Confidence Interval; LL = lower limit; UL = upper limit.

The only other significant interaction was the block × classification group × age group interaction. The investigation revealed that there were significant mixing-costs for all combinations of the age group and classification group factors (*p < 0.*001 in all cases) with the exceptions of the difference between the incompatible block and the mixed block incompatible no set-shifting trials for the CWS-Y and the CWNS-Y subgroups (Figure 2, Panel B). Two-tailed unpaired *t*-test analyses revealed that CWS-O had a larger set-shifting-cost than CWNS-O in their speed (slowed down more) from no set-shifting to set-shifting trials, *t*(35) = 1.83, *p* = 0.04, and also that CWNS-O had larger performance-cost than CWS-O when comparing their speed in the incompatible block with their speed for the no set-shifting trials of the mixed block, *t*(35) = 3.17, *p* < 0.005; all other differences in performance-cost between any subgroups were not significant (Figures 3, 4).

### Cognitive flexibility in terms of accuracy

#### Compatible trials

The coefficients for the multilevel logistic regression analysis are shown in Table 4. The model correctly classified 82.3% of the cases. The between-subjects effect for the classification group factor was not significant, *F*(1, 5,908) = 0.54, *p* = 0.46 (for CWS: *M* = 14.8%, SE = 2.3%; for CWNS: *M* = 17.4%, SE = 2.6%). The effect of age group was not significant, *F*(1, 5,908) = 1.79, *p* = 0.18 (for younger participants: *M* = 18.6%, SE = 2.7%; for older participants: *M* = 13.9%, SE = 2.3%). The only significant interaction was the one between age group and block, *F*(2, 5,908) = 8.18, *p* < 0.001. Investigation of this interaction revealed that both age groups made more errors in the mixed block for both the set-shifting trials (for younger participants: *M* = 23.7%, SE = 3.4%, *t*(5908) = −3.34, *p* < 0.005; for older participants: *M* = 20.3%, SE = 3.2%, *t*(5908) = −4.52, *p* < 0.001) and the no set-shifting trials (for younger participants: *M* = 23.2%, SE = 3.4%, *t*(5908) = −3.77, *p* < 0.001; for older participants: *M* = 22.2%, SE = 3.5%, *t*(5908) = −3.88, *p* < 0.001) compared to the compatible block (for younger participants: *M* = 11.2%, SE = 1.9%; for older participants: *M* = 5.4%, SE = 1.1%); there was no difference between no set-shifting and set-shifting trials of the mixed block for either the younger or the older group. Moreover, the two age groups differed in the compatible block, *t*(5908) = −2.64, *p* = 0.01, but not in the mixed block for either the set-shifting and the no set-shifting trials.

**Table 4:** Fixed coefficients for classification group, age group, and block for the compatible trials analysis of accuracy.

Model term	*B*	SE	*t*	*p*	Exp(*B*)	95% CI for Exp(*B*)	
						LL	UL
Intercept	−2.03	0.27	−7.47	< 0.001	0.13	0.08	0.22
Classification group (CWS)	−0.08	0.38	−0.20	0.84	0.93	0.44	1.96
Block (mixed-set-shifting)	1.04	0.16	6.40	< 0.001	2.84	2.06	3.91
Block (mixed-no set-shifting)	1.00	0.16	6.18	< 0.001	2.72	1.98	3.73
Age group (older)	−0.71	0.40	−1.77	0.08	0.49	0.22	1.08
Classification group (CWS) × Block (mixed-set-shifting)	−0.35	0.23	−1.48	0.14	0.71	0.45	1.12
Classification group (CWS) × Block (mixed-no set-shifting)	−0.19	0.23	−0.84	0.40	0.82	0.53	1.29
Classification group (CWS) × age group (older)	−0.15	0.58	−0.27	0.79	0.86	0.28	2.66
Block (mixed-set-shifting) × age group (older)	0.34	0.26	1.33	0.18	1.41	0.85	2.34
Block (mixed-no set-shifting) × age group (older)	0.55	0.25	2.18	0.03	1.74	1.06	2.87
Classification group (CWS) × Block (mixed-set-shifting) × age group (older)	0.78	0.38	2.08	0.04	2.19	1.05	4.59
classification group (CWS) × Block (mixed-no set-shifting) × age group (older)	0.07	0.37	0.20	0.84	1.08	0.52	2.25

The model included the random effect of an individual-level intercept. CI = Confidence Interval; LL = lower limit; UL = upper limit.

**Figure 5:**
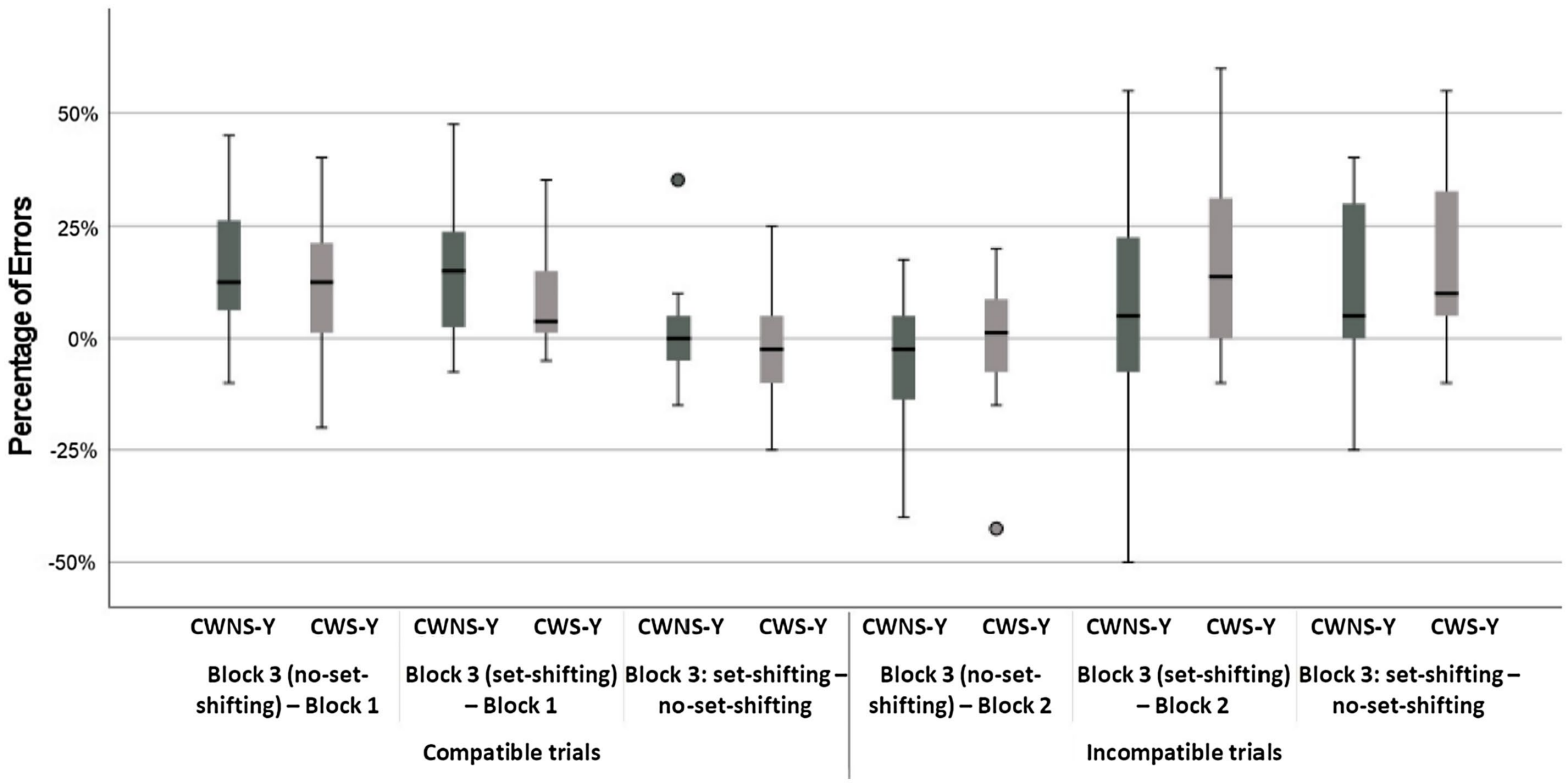
Boxplots of costs in errors for CWNS-Y and CWS-Y.

**Figure 6:**
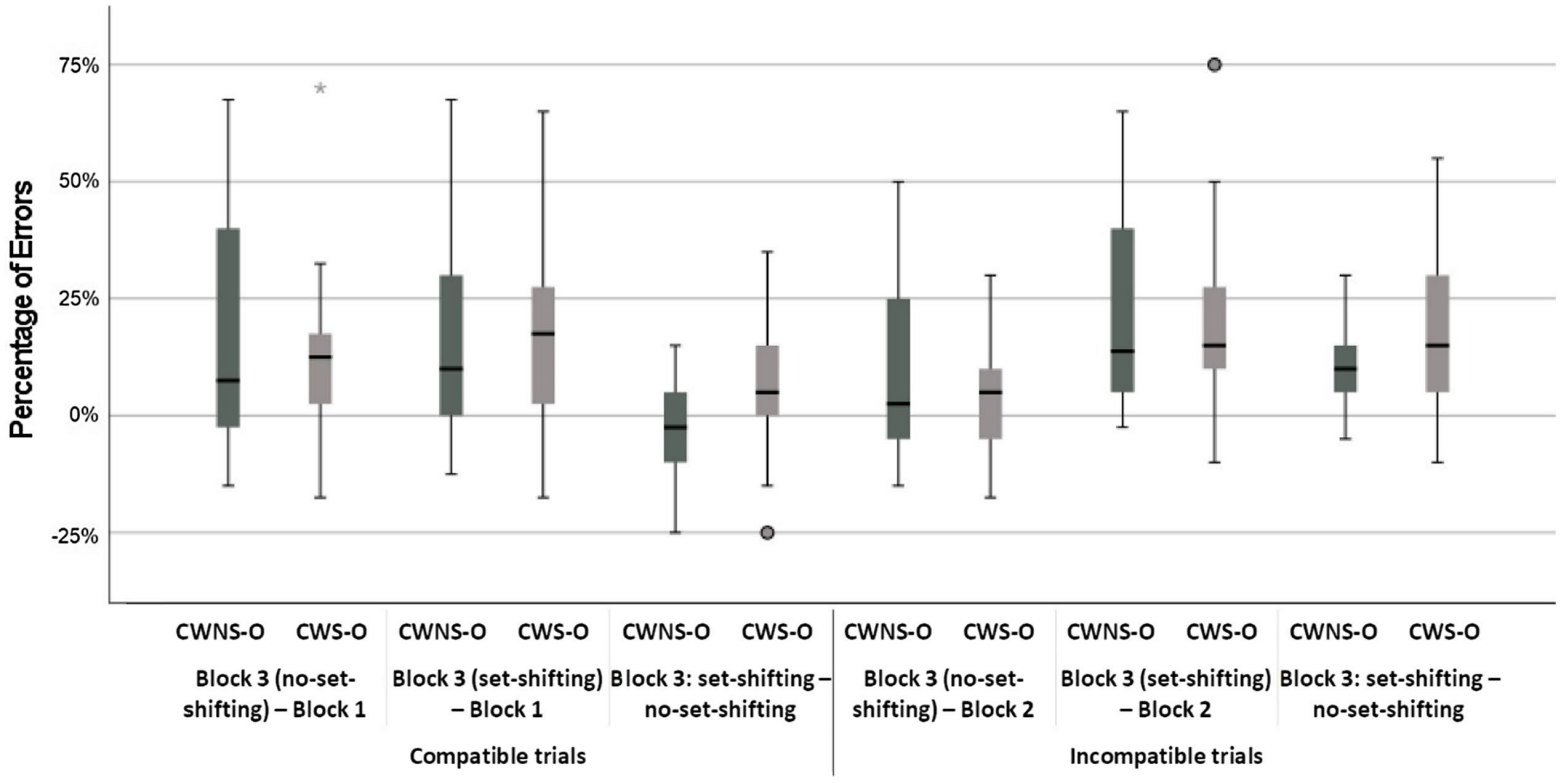
Boxplots of costs in errors for CWNS-O and CWS-O.

The classification group × age group × block interaction was not significant, but a simple main effects analysis revealed that all subgroups were less accurate in the mixed block both for the set-shifting and the no set-shifting trials (*p* < 0.001 in all cases), i.e., they exhibited significant set-shifting-cost; the difference between set-shifting and no set-shifting trials was not significant with the exception of CWS-O who made significantly more errors in set-shifting trials compared to no set-shifting trials, *t*(5908) = 2.05, *p* = 0.04 (i.e., they had significant set-shifting-cost; Figure 2, Panel C). Two-tailed unpaired *t*-test analyses revealed that the difference in performance-costs between any subgroups were not significant, with the exception of CWS-O who had larger set-shifting-cost from no set-shifting to set-shifting trials compared to CWNS-O, *t*(35) = 2.15, *p* = 0.02, and CWS-O had a significant increase in errors when they had to shift from set-shifting to no set-shifting trials compared to CWS-Y, *t*(35) = 1.98, *p* = 0.03 (Figures 5, 6).

#### Incompatible trials

The estimated marginal means for errors per group are presented in Table 5. The model correctly classified 74.7% of the cases. The between-subjects effect for the classification group factor was not significant, *F*(1, 5,908) = 0.39, *p* = 0.53 (for CWS: *M* = 23.0%, SE = 2.5%; for CWNS: *M* = 25.3%, SE = 2.7%). The effect of age group was not significant, *F*(1, 5,908) = 3.66, *p* = 0.06 (for younger participants: *M* = 27.8%, SE = 2.8%; for older participants: *M* = 20.7%, SE = 2.4%). The only significant interaction was the one between age group and block, *F*(2, 5,908) = 11.83, *p* < 0.001. Investigation of this interaction revealed that both age groups made more errors (i.e., mixing-cost) moving from the incompatible block to the mixed block, both for the set-shifting and no set-shifting trials (*p* < 0.001 in all cases; the only exception was the case of the difference between the mixed block no set-shifting trials and the incompatible block for the younger participants, *p* = 0.16); in addition, both age groups made significantly more errors for set-shifting trials compared to no set-shifting trials (for younger participants: *t*(5908) = 6.44, *p* < 0.001; for older participants: *t*(5908) = 5.76, *p* < 0.001). Moreover, the two age groups differed in the incompatible block, *t*(5908) = −3.67, *p* < 0.001, but not in the mixed block for either the set-shifting and the no set-shifting trials.

**Table 5:** Fixed coefficients for classification group, age group, and block for the incompatible trials analysis of accuracy.

Model term	*B*	SE	*t*	*p*	Exp(*B*)	95% CI for Exp(*B*)	
						LL	UL
Intercept	−0.89	0.21	−4.35	< 0.001	0.41	0.27	0.61
Classification group (CWS)	−0.44	0.29	−1.53	0.13	0.64	0.36	1.13
Block (mixed-set-shifting)	0.35	0.14	2.61	0.01	1.42	1.09	1.85
Block (mixed-no set-shifting)	−0.22	0.14	−1.57	0.12	0.80	0.61	1.06
Age group (older)	−1.09	0.30	−3.60	< 0.001	0.33	0.18	0.61
Classification group (CWS) × Block (mixed-set-shifting)	0.53	0.19	2.76	0.01	1.70	1.17	2.49
Classification group (CWS) × Block (mixed-no set-shifting)	0.16	0.20	0.78	0.43	1.17	0.79	1.75
Classification group (CWS) × age group (older)	0.58	0.43	1.35	0.18	1.79	0.77	4.18
Block (mixed-set-shifting) × age group (older)	0.95	0.21	4.54	< 0.001	2.58	1.71	3.88
Block (mixed-no set-shifting) × age group (older)	0.92	0.22	4.23	< 0.001	2.50	1.64	3.83
classification group (CWS) × Block (mixed-set-shifting) × age group (older)	−0.66	0.30	−2.21	0.03	0.52	0.29	0.93
classification group (CWS) × Block (mixed-no set-shifting) × age group (older)	−0.58	0.31	−1.86	0.06	0.56	0.30	1.03

The model included the random effect of an individual-level intercept. CI = Confidence Interval; LL = lower limit; UL = upper limit.

The classification group × age group × block interaction was not significant, but a simple main effects analysis revealed that there were significant performance-costs for all combinations of the age group and classification group factors (*p < 0.*001 in all cases) with the exceptions of the difference between the incompatible block and the mixed block incompatible no set-shifting trials for the CWS-Y, CWNS-Y, and the CWS-O subgroups (Figure 2, Panel D). Two-tailed unpaired *t*-test analyses revealed that no difference in performance-cost between any subgroups was significant (Figures 5, 6).

### Correlation between stuttering severity and mixing-, set-shifting-, and performance-cost

For the CWS, the relationship between the SSI-3 scores and the different types of costs in both response times and errors were investigated with Kendall rank correlation analyses. No significant correlations were detected for response times (*p* values between 0.76 and 1.00) or for errors (*p* values between 0.22 and 1.00).

## Discussion

In the present study, we investigated *CF* in CWS across a broad age range (4–10 years old) with the use of a single behavioral measure. Studies so far have either focused on younger (3–6 years old; Eichorn et al., 2018; Ntourou et al., 2018; Anderson et al., 2020) or older CWS (6–12 years old; Eggers and Jansson-Verkasalo, 2017; Piispala et al., 2017, 2018; Eichorn and Pirutinsky, 2021; Paphiti et al., 2022). Our results showed that: (a) all participants (of both age-groups) had longer response times for set-shifting trials compared to no set-shifting trials, (b) older CWS showed a larger set-shifting-cost than older CWNS, meaning that they required more time to go from no set-shifting to set-shifting trials and made more errors and, (c) older CWNS did not differ from younger CWNS in any of the speed or accuracy comparisons.

### Set-shifting takes time

It appeared that for all participant subgroups, set-shifting trials required more time than no set-shifting trials, as was the case in the Paphiti et al. (2022) submitted study (the only other study that used a mixed-block-design task to compare the speed of set-shifting trials to that of compatible, incompatible and of no set-shifting trials). Our first hypothesis was that CWS, CWS-Y, and CWS-O would be significantly slower compared to the corresponding CWNS group. This hypothesis was not supported. When we compared the compatible trials of the mixed block to those of the compatible block, there were no within groups or subgroups differences either in terms of speed or accuracy (i.e., no mixing-cost differences). Differences were revealed only when we conducted comparisons between the no set-shifting and the set-shifting trials (within-block comparisons). Set-shifting conditions rely on working memory to maintain both rules active and on inhibitory control to withhold the response to the rule that no longer applies (Garon et al., 2008; Schoemaker et al., 2013). The fact that all subgroups slowed down is in line with the available ANT literature (e.g., Eggers and Jansson-Verkasalo, 2017; Schuiringa et al., 2017). This is said to be indicative of the additional time needed for task-set reconfiguration, i.e., configurating the cognitive system for the new rules (Rogers and Monsell, 1995; Sohn and Carlson, 2000).

Our findings did not support the hypothesis that CWS would be slower compared to CWNS. Instead, they only showed a higher set-shifting-cost for CWS-O in terms of accuracy. They corroborate the findings of one of the two studies that used a mixed-block-design task that included participants of similar age range (6- to 10-year-olds; Eggers and Jansson-Verkasalo, 2017) with the CWS-O subgroup used in the present study. Our present findings are also in agreement with the Eggers et al. (2013) study on inhibitory control in 4- to 10-year-old CWS that showed CWS were unable to adjust their response style (slow down) to reduce the occurrence of errors. Being able to shift the emphasis from speed to accuracy or from accuracy to speed, is a strategy of pure control (Gopher et al., 2000). It is interesting that in the present study weaknesses were evident only in the older subgroup of CWS and not in the younger. Of course, caution is needed when interpreting our findings of comparing the compatible trials, as inhibitory control may have played a role in the outcome. Similarly, it is possible that the increase in errors in the Eggers and Jansson-Verkasalo study could be attributed to the effects of inhibitory control and results might have been different if a within-block investigation was conducted while, controlling for the effects of inhibitory control. Lastly, our results are partially in agreement with the findings of the Rocha et al. (2019) who reported that 7- to 9-year-old CWS performed slower and made more errors compared to controls.

Finally, no significant correlations were found between SSI-3 scores and the different types of costs. This means that there was no correlation between stuttering severity and the different types of costs found in performance or that, because of the variability of stuttering, our severity measure may not have been a good representation of overall stuttering severity. Even though in studies with CWS, it is a standard procedure to collect speech samples during data collection and not to include any recordings from the home, school, or social environment of the child for establishing stuttering severity, this practice may have influenced the findings.

### Set-shifting-cost is larger in older (but not in younger) CWS compared to CWNS

Our second hypothesis was that the differences (in speed) between the two groups and subgroups would be more evident when comparing the incompatible trials. This was supported only for CWS-O (Figure 2, Panel B). One of our findings showed decreased performance for the CWNS-O compared to CWS-O. CWNS-O slowed down more than CWS-O in the no set-shifting trials of the mixed block compared to the incompatible block, something that was unexpected. This cannot be attributed to the incompatible block being too easy for the CWNS-O, because both groups had similar speed and accuracy in this block. In the mixed block though, CWNS-O slowed down more possibly to avoid errors. Davidson et al. (2006) suggested that from the age of 6, children slow down more in these conditions to avoid errors (i.e., speed/accuracy trade-off) while most younger children fail to use this strategy (Zelazo et al., 2013). It is therefore possible that CWNS-O slowed down more in the mixed block to minimize the occurrence of errors.

The significant block × classification group × age group interaction revealed that CWS-O had larger set-shifting-cost (slowed down more) in set-shifting trials than in no set-shifting trials compared to CWNS-O. We believe that this is the most important and clear finding from the present study for two reasons: (a) mixed blocks are heavily demanding in terms of set-shifting and set-shifting correlates highly with *CF*; a clear measure of *CF* (Crone et al., 2006; Gajewski et al., 2010; Liu et al., 2016) and, (b) the within-mixed block comparisons of the incompatible trials allowed us to investigate set-shifting while controlling, at least to some extent, for the effects of inhibitory control. This finding is consistent with the findings of the Paphiti et al. (2022) study. In both studies there was a larger set-shifting-cost for CWS (CWS-O in the present study) in terms of speed. Our findings also corroborate the findings of the studies by Anderson et al. (2020), Eichorn et al. (2018) and Eichorn and Pirutinsky (2021) in which longer response times were reported for CWS. In the first two studies, the ability to switch was evaluated with younger participants (3.0–6.5 years old). In the latter study, older children participated (8.0–11.92 years old) and both switching and set-shifting were evaluated with the use of a modified version of the Dimension Change Card Sort Task in which a mixed block was included. These larger set-shifting-costs in CWS could be attributed to a speed-accuracy trade-off, or, in line with earlier assumptions (Eichorn et al., 2018; Anderson et al., 2020), to additional time needed by CWS to adequately process task demands. They could also be attributed to the fact that the CWS were trying to avoid errors, as they may perceive themselves as error-prone, something that was reported for adults who stutter (Brocklehurst et al., 2015).

*CF* has been associated with speech-language planning and execution. It allows speakers to detect errors prior to articulation but also to shift attention away from errors and stay focused on completing an utterance. From a theoretical perspective, the Vicious Circle Hypothesis (Vasić and Wijnen, 2005) suggests that speech disfluencies result from a faulty internal monitoring system. Slower performance in *CF* could mean that during speech-language production CWS might have more difficulty in directing their attention away from errors. Besides speech fluency, also other behavioral domains related to stuttering might be impacted. A slower performance in *CF* might also impact one’s behavioral responses when quick and complex responding are required in daily life (Schuiringa et al., 2017), such as responding to social cues and problem-solving (Brunnekreef et al., 2007; Schuiringa et al., 2017). *CF* was reported to play a role in emotional self-regulation and, therefore, weaknesses in *CF* may also trigger more stuttering in demanding communicative situations (e.g., time pressure, being interrupted; Eggers and Jansson-Verkasalo, 2017). Different authors have also hypothesized about the impact of lower self-regulation on the development of negative cognitions and anxiety in relation to stuttering (e.g., Eggers et al., 2022).

Taken all together, our findings indicated CWS-O compared to CWNS-O to have lower accuracy levels when no measures were taken for the effects of inhibitory control, and to have slower speed when measures were taken. It is of great importance that all the differences found between the two classification groups involved the older children, with CWS presented with lower performance. Although this was not a longitudinal study and it was not a direct evaluation of the causal mechanisms of developmental stuttering, it is possible that the weaknesses in *CF* contribute to stuttering persistence. It is more likely for CWS from the younger group to have spontaneous recovery, whereas it is less likely for CWS from the older group to recover. A possible explanation for the co-existence of weaknesses in *CF* and stuttering persistence could be due to a common underlying neurological mechanism. Nevertheless, it is of great importance, that all studies conducted on *CF* in CWS, reported similar findings, weaknesses in *CF* for CWS. The novelty of the present study was that it provided interesting insights about stuttering for both younger (4–6.91 years old) and older CWS (7–10 years old) while attempting to take into account the effects of inhibitory control. These findings along with those of the previous studies could possibly lead to a better understanding of this complex communicative disorder.

### Set-shifting may take longer to develop in CWS

In the area of developmental stuttering research, the findings of the Rocha et al. (2019) study suggested a possible delay in the maturation of *CF* for CWS, but no other study reported similar findings. In the present study, due to the large number of participants and the wide age group, we were able to make between age group comparisons.

At first glance, descriptive statistics indicated improvement in both speed and accuracy for the older groups, but the statistical analyses revealed no differences between the younger and older CWNS in speed or accuracy. For the CWS subgroups, the statistical analyses revealed that CWS-O compared to CWS-Y were faster for the no set-shifting trials and had a higher increase in errors when moving from set-shifting to no set-shifting trials. Given that this was a cross-sectional study and given that no statistically significant differences between the younger and the older participants were found under set-shifting task conditions, we cannot claim that the present study provided us with evidence that *CF* develops differently for CWS. A longitudinal study would have provided us with insights concerning the possibility of a parallel or delayed development of *CF* for CWS and CWNS. Therefore, further research is needed with the use of a longitudinal study design.

## Conclusion and future directions

In sum, the results of the present study show a higher set-shifting-cost (increased slowing down and more errors) for the older CWS (compared to the older CWNS) and partly corroborate earlier research findings with CWS of similar age. All participants required more time for set-shifting trials. Our findings suggest possible (a) *CF* weaknesses for CWS (7–10 years old) and (b) associations of these weaknesses to stuttering persistence. Further investigation is needed into the underlying mechanism of how weaknesses in EF could play a role in stuttering persistence and in the presence of speech disfluencies reported in non-stuttering populations (e.g., Engelhardt et al., 2010).

## Data availability statement

The datasets generated for this study will be made available by the authors, on request to the corresponding author.

## Ethics statement

This study was approved by the Research Ethics committee of Leuven University Hospitals. Parents signed an informed consent form.

## Author contributions

KE designed the study and collected the data. MP analyzed the data and wrote the first draft of the manuscript. Both authors contributed to the article and approved the submitted version.

## Funding

Data collection was supported by Thomas More’s PWO research fund.

## Conflict of interest

The authors declare that the research was conducted in the absence of any commercial or financial relationships that could be construed as a potential conflict of interest.

## Publisher’s note

All claims expressed in this article are solely those of the authors and do not necessarily represent those of their affiliated organizations, or those of the publisher, the editors and the reviewers. Any product that may be evaluated in this article, or claim that may be made by its manufacturer, is not guaranteed or endorsed by the publisher.
